# Supplementary material for: A Narrative Inquiry into the Practices of Healthcare Workers’ Wellness Program: The SEED Experience in New South Wales, Australia
Source: Int J Environ Res Public Health. 2022 Oct 13;19(20):13204. doi: 10.3390/ijerph192013204 (PMC9603759; doi:10.3390/ijerph192013204)
Supplement: Supplementary file 1 [file ijerph-19-13204-s001.zip › ijerph-1889354-supplementary.pdf]

Table S1. A Selection of Qualitative Interview and Focus Groups Questions by Topic.

Topic 1: Experiences with South Coast bushfires

A. Can you describe your experience of the South Coast bushfires?
B. What involvement have you had with SEED's response to the bushfires?
C. How has SEED response to the bushfires evolved?
D. If SEED did not exist, what impact would the bushfires have on the hospital staff?
E. If you were to provide advice to other rural and regional communities in relation to the practices that are most effective in supporting mental health, healing and recovery post cumulative disasters, what would you recommend?

Topic 2: Experiences with COVID-19

F. Can you describe your experience of the COVID-19 lockdown?
G. What involvement have you had with SEED response to the COVID-19 lockdown?
H. How has SEED response to COVID-19 lockdown evolved?
I. If SEED did not exist, what impact would COVID-19 lockdown have on the hospital staff?

Topic 3: Philosophy and practices of SEED

J. What does SEED look like in your workplace?/ What programs and activities did it initiate?
K. How would you describe SEED's philosophy?/ What is SEED about?
L. What values does SEED encourage and promote?

Topic 4: Personal and professional impacts of SEED

M. Has SEED impacted you personally in any way?
N. Do you think SEED impacted your workplace experience in any way?
O. For leaders: Do you think SEED impacted your leadership in any way?

Note. This table reflects a sample of questions asked to healthcare workers and leaders. The use of the guide was intended to be flexible to follow the ebbs and flows of the conversation. Probes focused on eliciting specific examples, and the contexts within which challenges and strengths emerged.

Table S2. Identified Themes and Corresponding Codes.

Themes	Codes
1. Responsive and compassionate leading	1. Recognition of crisis by leaders<br>2. Immediacy of action to address the psychological needs of staff<br>3. Ongoing management support<br>4. Permission from leaders to engage in wellness activities<br>5. Normalising focus on wellness in a workplace<br>6. Role modelling practices of kindness, care, and compassion<br>7. Being recognised and valued by the leaders
2. Co-designing wellness activities with staff	8. Co-design of SEED activities<br>9. Sense of ownership of SEED activities at each site<br>10. Consultation with staff at each stage<br>11. Everyone is invited to SEED space<br>12. Development and meaning of SEED acronym and symbol<br>13. Doing things differently to the usual structures and processes of health system
3. Listening to understand	14. Holding space for one another<br>15. Learning and practicing deep listening<br>16. Willingness to accept not having answers<br>17. Staying present and not jumping to solutions<br>18. Sitting in stillness
4. Creating a safe and healing space	19. Healing from the trauma of bushfires and other traumatic experiences<br>20. Helping staff recognise and process their emotions<br>21. Allowing oneself and others to show vulnerability<br>22. Non-judgemental space<br>23. Sharing stories<br>24. Time and space for self-reflection<br>25. Being your authentic self in the workspace<br>26. Being heard while expressing your needs<br>27. Permission to self to incorporate wellness into the workspace
5. Connecting with others	28. Getting to know each other better<br>29. Focus on the common humanity and the human experience<br>30. Different manifestations of acts of kindness towards each other<br>31. Friendships made in the workplace<br>32. Connecting through the shared experience
6. Collective caring	33. Different manifestations of staff showing caring for each other<br>34. Companioning: being there for each other<br>35. Giving and receiving compassion from colleagues<br>36. Seeing and acknowledging the strengths, efforts, and contributions of others<br>37. Giving and receiving empathy from colleagues<br>38. Learning and practicing respecting each other in workplace<br>39. References to giving and receiving trust<br>40. Learning how to validate others and their experiences<br>41. "People first" - putting people before process

	42. Developing a collective staff identity within the local health district
7. Diversifying and localising wellness activities	43. SEED is adaptable to fit the needs of staff at each site<br>44. SEED practices/activities are creative and/or sparking creativity in staff<br>45. SEED practices/activities are developing/evolving naturally to suit the needs at any given time<br>46. Material arrangements that SEED brought to workspace, e.g., the quiet room, cards, food trucks<br>47. The vast array of SEED activities to select from and/or create new ones<br>48. References to the Wellness Champions and their role in embedding/sustaining SEED
8. Striving for sustainability	49. References to challenges and barriers to introducing and sustaining a wellness program in healthcare settings<br>50. Plans for how to sustain SEED over time<br>51. Striving for a culture shift within the workplace<br>52. References to SEED scaling up and expanding to other sites
